# Histological and electrophysiological evidence on the safe operation of a sharp-tip multimodal optrode during infrared neuromodulation of the rat cortex

**DOI:** 10.1038/s41598-022-15367-4

**Published:** 2022-07-06

**Authors:** Á. Cs. Horváth, S. Borbély, F. Mihók, P. Fürjes, P. Barthó, Z. Fekete

**Affiliations:** 1 Research Group for Implantable Microsystems, Faculty of Information Technology and Bionics, PPKE, Budapest, Hungary; 2 Sleep Oscillations Research Group, Institute of Cognitive Neuroscience and Psychology, RCNS, ELKH, Budapest, Hungary; 3 Neuronal Network and Behavior Research Group, Institute of Experimental Medicine, ELKH, Budapest, Hungary; 4 Department of Control Engineering and Information Technology, BUTE, Budapest, Hungary; 5 Microsystems Laboratory, Centre for Energy Research, ELKH, Budapest, Hungary

**Keywords:** Biomedical engineering, Neurophysiology

## Abstract

Infrared neuromodulation is an emerging technology in neuroscience that exploits the inherent thermal sensitivity of neurons to excite or inhibit cellular activity. Since there is limited information on the physiological response of intracortical cell population in vivo including evidence on cell damage, we aimed to create and to validate the safe operation of a microscale sharp-tip implantable optrode that can be used to suppress the activity of neuronal population with low optical power continuous wave irradiation. Effective thermal cross-section and electric properties of the multimodal microdevice was characterized in bench-top tests. The evoked multi-unit activity was monitored in the rat somatosensory cortex, and using NeuN immunocytochemistry method, quantitative analysis of neuronal density changes due to the stimulation trials was evaluated. The sharp tip implant was effectively used to suppress the firing rate of neuronal populations. Histological staining showed that neither the probe insertion nor the heating protocols alone lead to significant changes in cell density in the close vicinity of the implant with respect to the intact control region. Our study shows that intracortical stimulation with continuous-wave infrared light at 1550 nm using a sharp tip implantable optical microdevice is a safe approach to modulate the firing rate of neurons.

## Introduction

The therapy and treatment of neurodegenerative diseases is gaining more attention due to the aging society^1^. Stimulation of particular brain regions is available by electrical, optical, mechanical and chemical means^2^. Infrared neuromodulation (INM) is a thermal stimulation technique, which involves the contribution of several overlapping fields like transcranial photobiomodulation (tPBM), transcranial near-infrared laser therapy (NILT) or low-level laser therapy (LLLT). All these have great potential both in the peripheral and central nervous system^3^. Irradiation of intact neurons with near-infrared light was effectively used to modulate cellular activity both in vitro^4,5^ and in vivo^6–9^ and either using pulsed mode, high-power illumination^7,10^ or continuous-wave, low-power illumination^5,8,9^. Induced temperature gradient (dT/dz or dT/dt) or background temperature elevation of the tissue is required for pulsed or continuous wave IR light to generate an action potential (AP).

The key advantage of INM is that it does not need any genetic modification of the target cells, and can be applied in a spatially confined region without causing electrical artifacts in electrophysiology^8^. The governing mechanism of INM is of photothermal origin, as it was found that water molecules are acting as the chromophores which transform photon energy to heat within the tissue^11^. There are multiple mechanisms in the cells contributing to this modulatory effect. Temperature sensitive ion channels, dominantly the transient receptor potential vanilloid (TRPV) ion channel family discovered by the 2021 Nobel laureates Michael J Caterina and David Julius^12^, are inherently sensitive to the changes of absolute temperature in the extracellular space^13^. Shapiro et al. has experimentally proven that electrostatic changes in the cell membrane due to temperature change are also responsible for elevated activity of neural cells^11^.

Besides the excitatory effects, inhibition of neural activity was first presented by Cayce et al.^14^. Later, suppression of electrically initiated AP generation and propagation was also observed in nerve preparations^15^. The effect of the elevated baseline temperature was hypothesized in the background of the mechanism^16^, however, a more recent hypothesis claims that an increase in temperature leads to a net increase in the hyperpolarizing currents actually overcoming depolarizing currents^17^. Ganguly et al. proved that potassium channel activation has a major role in the blocking of action potential propagation in *Aplysia californica* nerves, while sodium channels play no significant role in the thermal response. Using a nanoengineered implantable photonic device, an optrode, our group was the first who demonstrated in the cortex and hippocampus of rats that infrared modulation of the multi-unit activity can be either excitatory or inhibitory depending on the targeted cellular layer^8^.

Although a number of studies demonstrated the efficacy of infrared stimulation, only a few attempts have been made to prove the safety of this neuromodulation approach. The extent of cell loss in response to the stimulation through the intact surface has been investigated using pulse-mode operation^9,18,19^. However, the temporal parameters like wavelength, frequency and length of the pulse train varied among experiments, a threshold between 0.4–1 J/cm^2^ was determined using optical fibers. Despite these promising results there is still a lack in histological data on the effect of intracortical infrared stimulation. In the case of intracortical stimulation it is reasonable to combine optical stimulation device with neurophysiological technique.

To reduce cell damage, one potential opportunity is to integrate several functionalities on a single device, which has been demonstrated by a few microengineered solutions^20–22^. This way the parallel insertion of multiple probes can be avoided, which would inherently correspond to cell death along the probe tracks. In our past experiment, a silicon microneedle acting as an infrared waveguide and holding multiple recording sites, and temperature sensor has been proposed. Even though, our study confirmed that such a blunt tip optrode can be efficiently used to optically modulate and electrically interrogate cellular activity in vivo, and changes in neuronal spike rate was found reversibly after several trials, there is still room to improve the efficiency and safety of this device^8^. Boros et al. found that such an optrode has a 32% overall IR waveguide efficiency with a simple blunt ending^23^ which can be further improved with an additional 17% by modifying the tip ending to a sharp shape^24^. According to these findings, symmetrical sharp tips between 14° to 60° vertex angle (what was referred to as 7° to 30° tip angle α) provide better waveguide efficiency than the blunt version (180°). The more pointed types within this region (< 30°) anticipate similar local heating effects like the blunt version. Tip shape of an optrode further influences penetration force and tissue dimpling during implantation^25^. By dimpling of brain tissue capillary vessels can be squeezed to such an extent that may cause irreversible damages to the neurons. So, a sharp tip optrode version contributes to a better yield during recording the activity of individual neurons. First, this modification seems an easy change in the geometry without any necessary changes in the micro- and nanomachining processes, however, the characteristics of heat distribution will necessarily change considerably due to the change in the optical pathway. In our paper, we demonstrate and characterize a novel, sharp tip multimodal optrode microdevice.

Besides describing the change in thermal properties, further in vivo experiments have proved that suppression of the neural activity in the infragranular layer of the rat somatosensory cortex can be achieved. Since potential cell death in response to continuous-wave infrared illumination in the deep tissue is barely investigated in the literature, we also give histological evidence on the effect of continuous-wave infrared illumination at 8 mW on the survival of cells.

## Results

### Electrochemical properties

**Figure 1:**
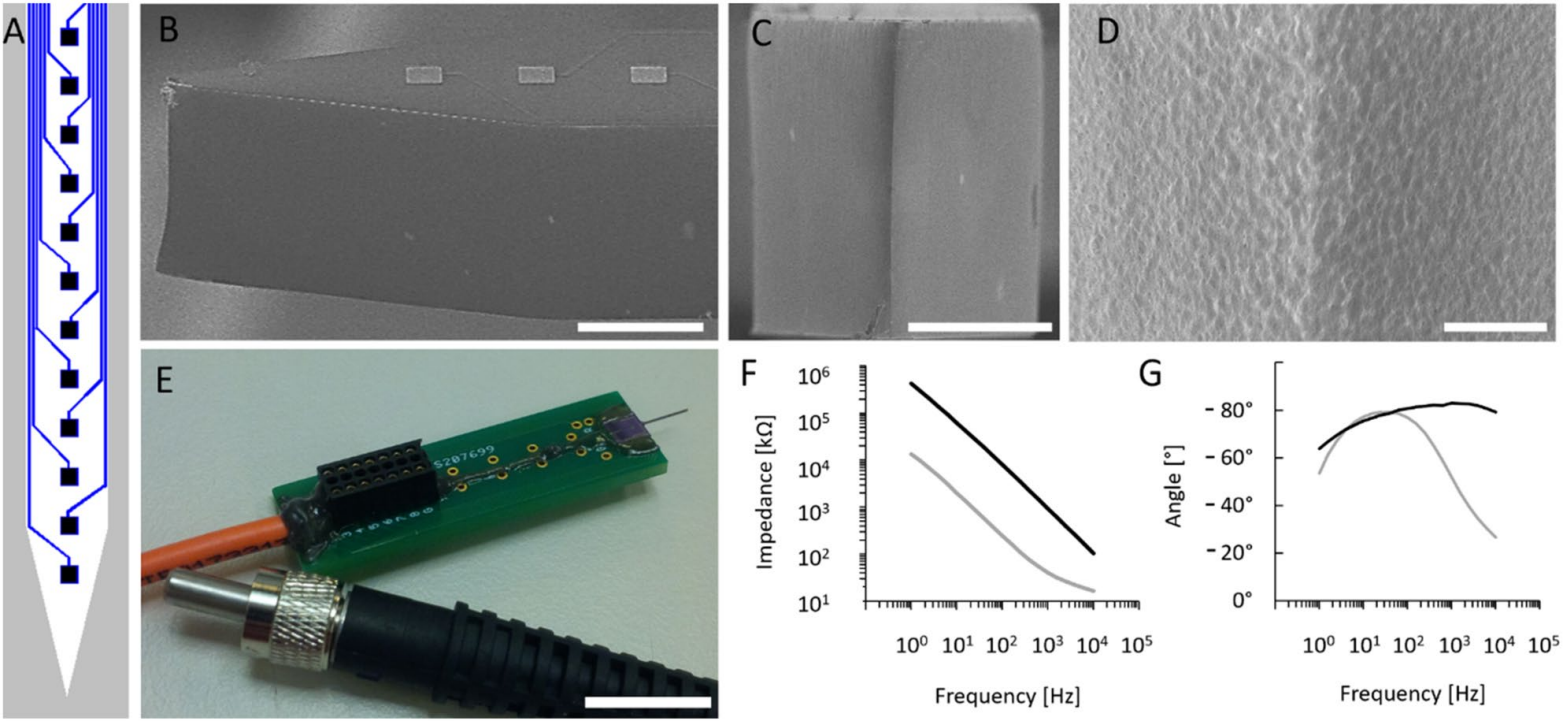
Topology of the linear array of recording sites along the probe shaft (**A**). Scanning electron microscopy images on the probe tip: sideview (**B**), front view (**C**) and close view (**D**) showing surface quality of the outcoupling region of the optrode chip. Assembled stimulation device (**E**). Representative Bode plot showing the impedance magnitude (**F**) and phase (**G**) with respect to frequency. Black line shows the initial condition after the micromachining processes have been finished, and the grey line shows the final condition after electrodeposition of porous platinum is completed. Scale bars represent 100 µm on (**B**) and (**C**), 30 µm on (**D**) and 10 mm on (**E**).

The electrical performance of the Pt recording sites was tested by electrochemical impedance spectroscopy (EIS) which is a widespread method in this field^26–31^. The amplitude and phase characteristics of the impedance of one selected Pt site before (black) and after (grey) porous Pt electrodeposition are shown in Fig. 1F,G in case of a representative optrode. Impedance magnitude at 1 kHz was collected from the investigation of four optrodes with 12 sites on each. Average impedance magnitude at 1 kHz of the sites within individual optrodes was derived from the mean value of the four averages as 977.8 ± 80.5 kΩ before and 89.6 ± 43.1 kΩ after electrodeposition. Impedance at 1 kHz is a widely used measure of electrode patency^32^. In the literature, the impedance of in vivo recording electrodes at 1 kHz typically range between 50 kΩ–1 MΩ^27^. Although the impedance at 1 kHz of the flat recording site (before electrodeposition) can be considered as sufficiently low for many in vivo applications, which aims to record the activity of single neurons, reduced impedance in case of b-Pt coverage further improve thermal noise properties of the electrodes, which is proportional to the square root of the real part of the impedance^33^.

### Heat-distribution

**Figure 2:**
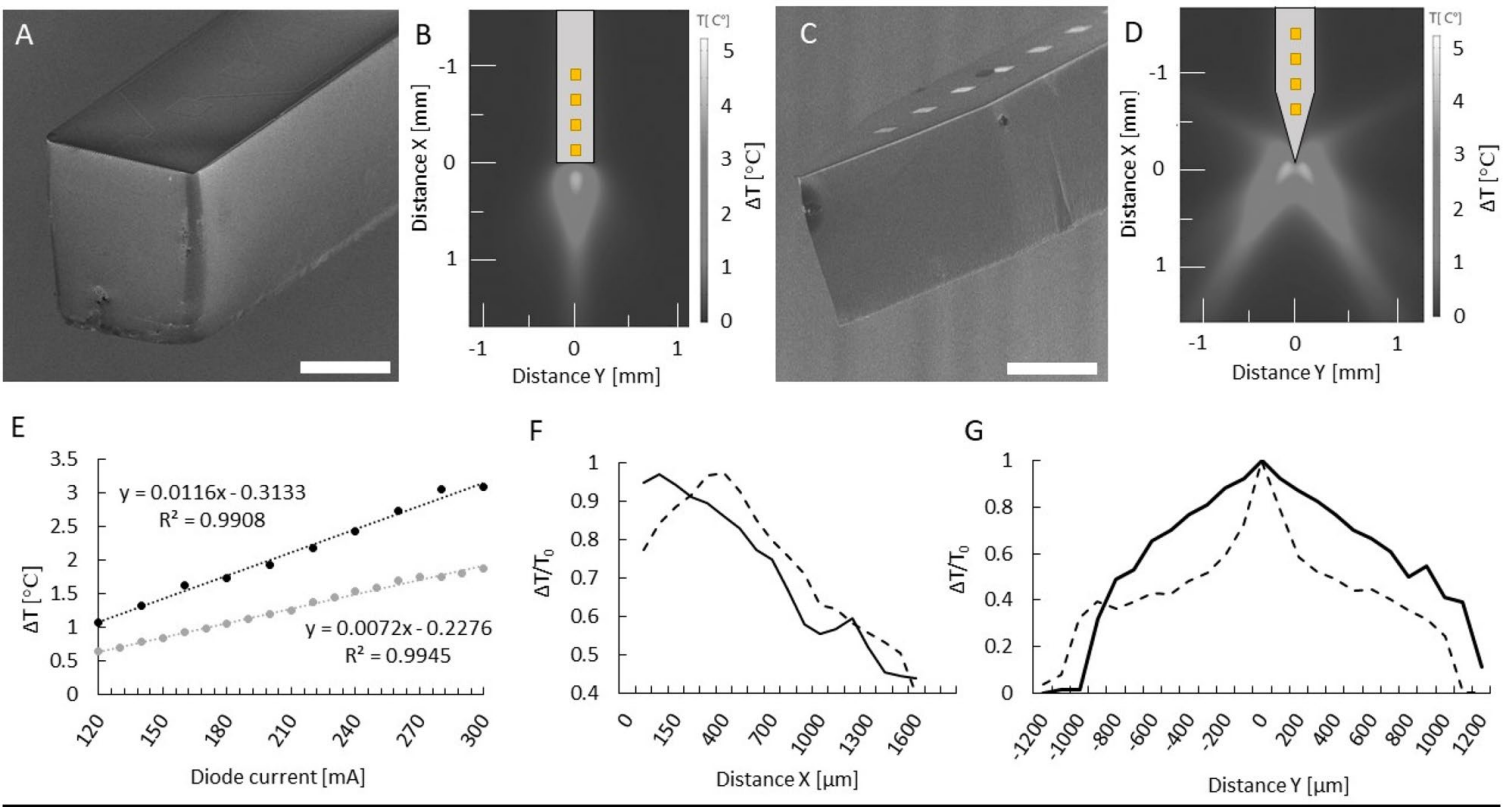
(**A**,**C**) Scanning electron microscopy view of different optrode tip versions. (**B**,**D**) Heat distribution based on the simulation of Boros et al.^24^. The grayscale colormap shows temperature differences in the °C range. (**E**) Representative calibration curve of a sharp (black) and a blunt (grey) tip optrode. (**F**) Axial decay of heat for sharp (normal line) and blunt (dashed line) tip optrodes. (**G**) Distribution of heat from along the axis Y perpendicular to the shaft at x = 200 µm from the tip of sharp (normal line) and blunt (dashed line) device. Scale bars are 100 µm in both (**A**,**C**).

Figure 2B,D show a simulation result of heat distribution in the brain tissue near the tips of a blunt and a sharp tip optrode, respectively (based on the simulation results of Boros et al.^24^). Figure 2A,C present the scanning electron microscopy (SEM) views of optrode samples with each tip version. Regarding the achievable maximum temperature, both tip endings anticipate similar local heating effects. However, it must be pointed out that the shape of the thermally affected regions is different which suggests that the amount of thermally affected neurons can also be changed. Figure 2F,G compare the normalized distribution of optically induced heating along the two axes around the optrode’s tip during the above mentioned measurements in water medium (cf. Fig. 2a in^8^). These results harmonize well with simulation. In Fig. 2F it can be observed that the place of maximum of heating is 300 µm closer to the sharp tip than in case of the blunt. The reasons for this difference are attributed to different tip shape: on one hand according to the simulation of Boros et al.^24^ most of the light intensity exits near the vicinity of the tip which consists of less material than the blunt version, while in the case of the sharp tip, therefore the cooling effect of the silicon material as a heat-conductor has weaker performance. On the other hand, those many rays which are emitted from the tilted sidewalls of the sharp tip have weaker effect on heating the region just facing the midline of the axis of the shaft causing the phenomenon that in this midline direction the depth of remarkable heating is shallower. Also, note that the distance between the recording site closest to the tip and the place of maximum of heating is nearly the same in the two cases.

Although the blunt optrode version produces a place of maximum of heating further, its closest site is less than 10 µm away from the edge of the silicon shaft while the closest site of the sharp optrode version is placed around 200 µm away from the sharp peak of the silicon shaft. In Fig. 2G. the difference in properties of the distribution of optical heating along the direction perpendicular to the shaft is more remarkable. The full width at half maximum are 0.7 mm and 1.6 mm for the blunt and sharp version, respectively, representing the wider effective cross-section. In short, the more divergent beams of sharp optrode heat an area that spreads at a similar depth but wider in space to a similar temperature than in the case of a blunt tip. Based on these results, it should be noted that neurons monitored through the integrated recording sites are not located in space, where the maximum change of temperature is induced. Instead, the interrogated neurons are exposed to about 60–80% of this value.

### Suppression of neural activity in vivo

**Figure 3:**
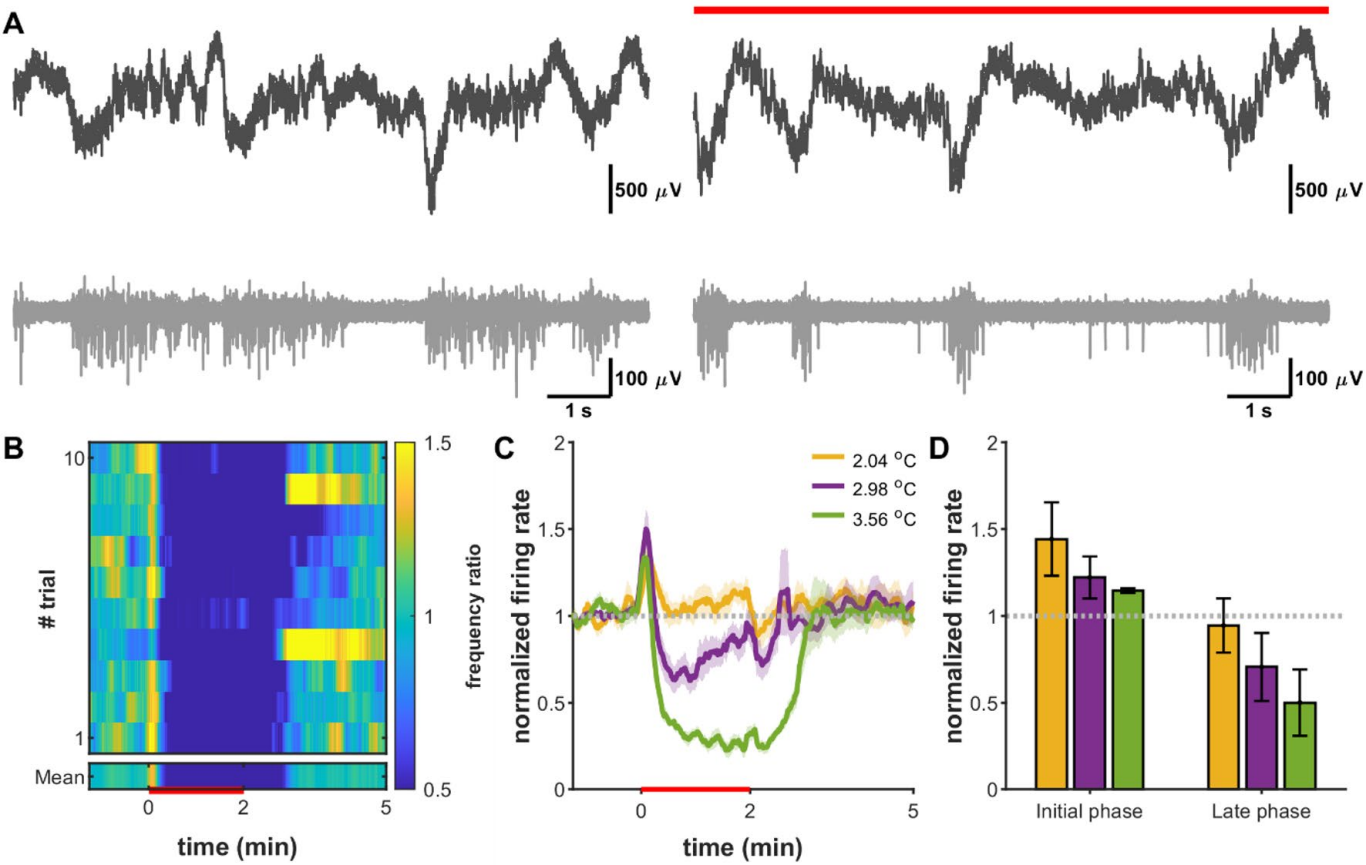
Infrared light induced suppression of cortical neurons. (**A**) Raw local field recording (black) and multiunit trace (gray) before (left) and during (red line) a heating session beyond the initial phase. (**B**) Average normalized spike rate across 15 trials (heat map) in case of 3.56 °C heating. (**C**) Normalized mean firing rate traces based on multiunit activity. (**D**) Cumulative normalized firing rate results from the effect of different heating protocols in the early (0–15 s) and late (48–120 s) phases of heating.

Since the optical performance of our optrode shaft acting as a bulk waveguide is inherently influenced by the tip, it was necessary to test the efficacy of modulating the neural activity. In this section, the electrophysiology results of three animals are detailed. The recorded local field potential data was band-pass filtered between 400 and 7000 Hz, then multi-unit activity was extracted from the filtered signal, based on thresholding method. Representative local field potential and multi-unit activity is shown in Fig. 3A. Signal-to-noise ratio of optrode recording was sufficient to follow the influence of infrared irradiation to the activity of neuronal population. These raw data traces confirm that this type of optical neuromodulation achieved by the introduced optrode with the applied longer wavelength (1550 nm) does not produce photoelectric artefact in the metal (Pt) electrode recordings.

The multi-unit activity was used to analyze the general cell firing rate changes of local cortical networks. Cortical neurons in the supragranular layers were sensitive to local temperature increase and showed a short onset paroxysmal activity followed by steady-state inhibitory response in the applied range of optical powers (Fig. 3B,C), which proved to be dependent on the temperature change (Fig. 3C,D). While Fig. 3B,C show representative data from a particular animal that proves the efficacy of various heating protocols, Fig. 3D contains the change in firing rate averaged from all animals.

The firing rate decreased gradually parallel with temperature rising, which showed slow recovery after the end of heating sessions. These findings are in agreement with our previous recordings using a blunt tip optrode device for suppressing the firing rate in vivo^8^, which confirms that sharp-tip optrodes holding embedded infrared waveguides effectively delivers and couples infrared light into the stimulated region around the probe tip. Supplementary Figure S1 shows further examples of infrared light induced suppression of cortical neural activity in four in vivo experiments different from Fig. 3A–C. Those data were also taken into account in the calculations of Fig. 3D.

### Histology

We used NeuN immunocytochemistry to visualize surviving cells of the somatosensory cortex in the proximity of our probes. High resolution digital images of these sections made the quantitative cell count analysis possible. Neurons were automatically distinguished from background by our custom written Mathworks Matlab based script, then manual verification of detection was made. Two-dimensional location data of cells were summarized in regions of interests (ROIs) defined at the probe insertion sites and at cortical regions high distant from injuries. The latter one was used as reference.

**Figure 4:**
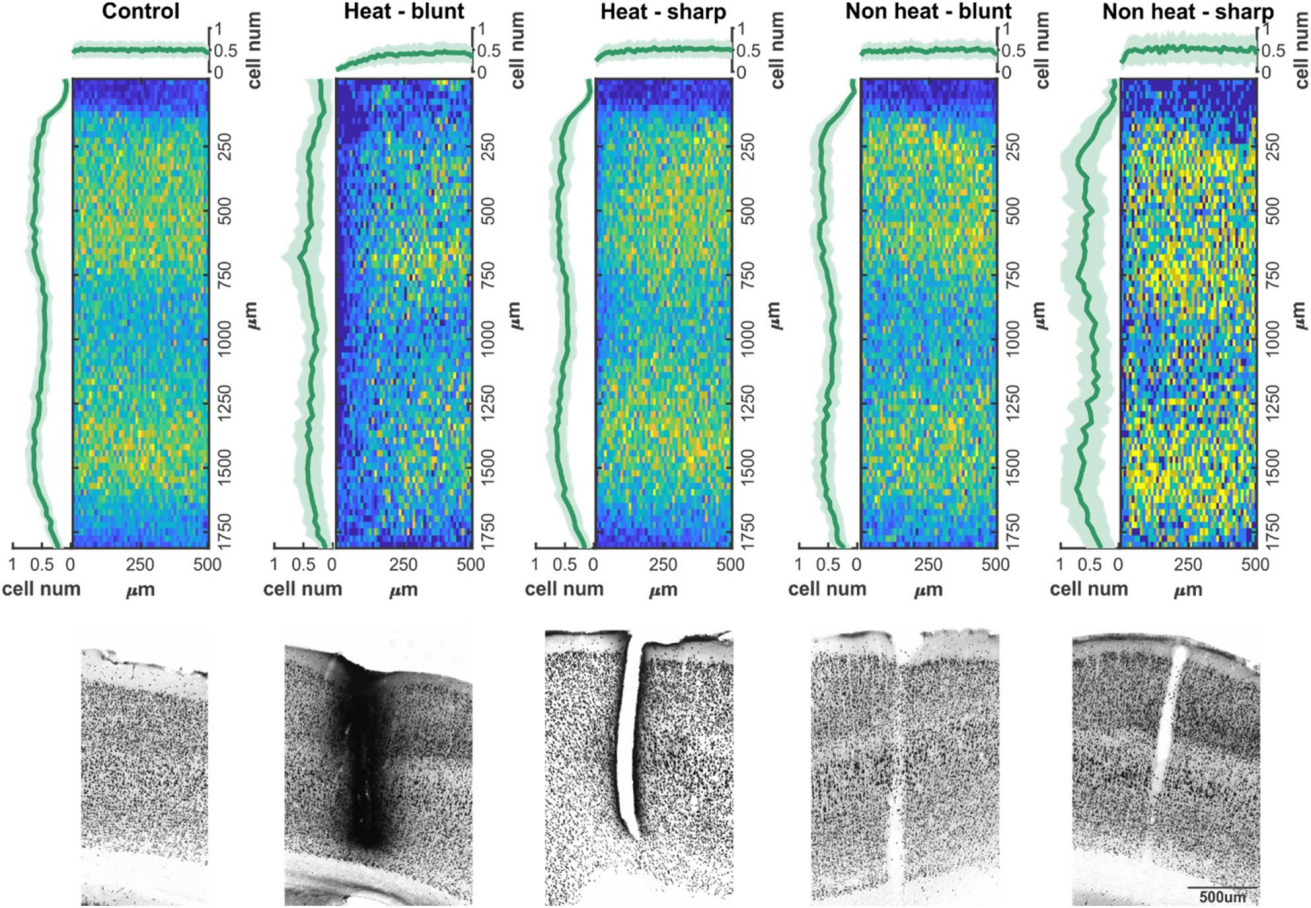
Cell density profiles and two-dimensional distribution of neurons. Top (colored) line: two-dimensional averaged histograms of NeuN immunostained sections from regions of interest in control and probe disrupted cortical areas. Vertical plots show mean cell density as a function of cortical depth, while horizontal traces show mean cell density as function of electrode distance (or in control sections the edge of region of interest). Bottom (grayscale) line: Characteristic examples of NeuN sections for each experimental group.

**Figure 5:**
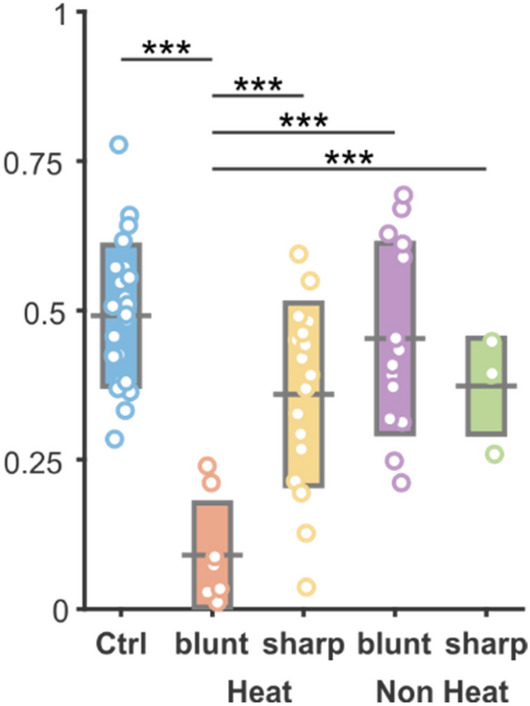
Cell number changes in each experimental group. The mean number of cell counts in each group. Black bar represents mean value, rectangles show standard deviation of mean, while open circles are individual cell count data. Horizontal black lines and stars show the level of significance in a comparison of a given pair of datasets. ***Symbol represents p < 0.001.

According to the cell distribution data (Fig. 4), control regions of somatosensory cortex did not show any change in mean number of cells as a function of distance from the edge of ROI, while on vertical axis two local maxima were found in the neuronal density (between 250–750 µm and 1300–1600 µm). The heating protocols or the probe insertion had no effect on cortical depth profile of cell count in either case, however, the neuronal density was reduced within 75 µm distance (Figs. 4 and 5), if a blunt electrode was inserted in an infralight heated experiment. Sharp electrodes, and blunt electrodes without heating had no effect on neuronal density at any distances (Figs. 4 and 5). A potential explanation for the elevated cell death in case of heating with blunt tip optrode may be the higher radiant exposure compared to sharp-tip devices. It is also possible that cell membranes exposed to higher shear forces along the probe track during device insertion are more sensitive to changes in extracellular temperature. Such hypotheses should be addressed in future studies on deep tissue infrared stimulation, particularly because extracellular recordings along the optrode shaft solely did not indicate cellular damage.

## Discussion

In our work, a sharp tip implantable optrode consisting of a bulk waveguide and electrophysiological recording sites was used to suppress the activity of neurons in the rat somatosensory cortex with spatially localized continuous-wave infrared illumination. Our current work provides evidence that a sharp-tip needle-like photonic microdevice is a feasible mean to reproduce and go beyond the recent results of the field of INM without the need to implant multiple devices to stimulate and record neuronal activity.

Immunostaining of brain tissue affected by IR stimulation revealed that the heating protocols or the probe insertion had no significant effect on cortical depth profile of cell count.

The first in vivo evidence on the efficacy of suppressing the firing rate of neurons was demonstrated by Cayce et al. in the rat somatosensory cortex^14^. Limitation of their approach was the application of a large spot size (850 µm) illumination with an optical fiber positioned above the exposed cortex. The penetration depth if pulsed IR light (ƛ = 1,850 nm) estimated to be around 300–600 µm in the tissue facilitated the stimulation of inhibitory neurons mostly located in layer II. Recording of evoked activity performed within the first 500 microns of the cortex with a Tungsten microelectrode revealed that decrease in spike rate due to the exposure to IR pulse trains (repetition rate = 100 Hz, pulse train duration = 500 ms, pulse width = 250 μs, radiant, exposures: 0.043 J/cm^2^ and 0.12 J/cm^2^) is repeatable. As found in our study in layer V-VI (Fig. 3B,C and Supplementary Figure S1) in the rat somatosensory cortex, suppression was also maintained for a longer period (more than 1 s) after stimulus onset. However, their work not highlighted, the initial phase of the stimulation onset also induced a short-lasting excitation period when using continuous-wave illumination (Fig. 3D). A more recent work of Wang et al. stimulating (ƛ = 980 nm, repetition rate = 300 Hz, pulse train duration = 500 ms, pulse width = 100–400 μs, radiant, exposures: 0.268 to 1.071 J/cm^2^) the surface of the cortex (tip of a 105 µm core-diameter optical fiber placed above the cortex at 7,00–1,000 microns) and measuring the evoked potentials with a Tungsten electrode already in layer V of the motor cortex, and found similar patterns in the change of spike rate, even though the applied wavelength resulted in lower absorption and therefore a different stimulation profile^34^.

Our prior work on the local stimulation in the deep cortical regions (1,300 µm) and in the hippocampus of rats revealed first that in vivo response to continuous wave infrared irradiation (ƛ = 1,550 nm) exhibit cell-specific patterns^8^, and is tunable with energy density as also proved by Xia et al. in cultured rat cortical neurons^5^, and Wang et al. in vivo^34^, in case of continuous and pulsed wave illumination, respectively. Regarding the rate of suppression, our findings, in agreement with the work of other studies^5,14^, confirms that the relative change of the tissue temperature governs the inhibitory activity, but full block of the neural activity cannot be achieved in vivo if staying within our illumination parameter space.

Besides the stability and repeatability of stimulation patterns, introducing a new technical approach also requires the assessment of cell damage around the stimulation device. Using a sharp-tip optrode with an embedded waveguide has clear advantages over blunt tip probes or fibers by reducing either penetration forces or dimpling during the insertion process^25,35^. Besides the influence of device dimensions on neuronal survival, the effect of continuous-wave infrared irradiation with our optrode in the deep tissue must be also checked independently. A number of histological studies in the literature has investigated the thermal damage threshold of pulsed infrared stimulation^3^. Radiant exposure, repetition rate and maximum duration of stimulation were found to be the key parameters when applying pulse trains for a longer period of time to the rat sciatic nerve^6^. Goyal et al. demonstrated that no functional change is caused in the guinea pig cochlea after exposing to 5 h long IR stimulation (ƛ = 1,869 nm) with 100 µs pulse (radiant energy less than 25 µJ/pulse) duration at 250 Hz^36^. Rat sciatic nerve exposed to metal nanoparticle mediated thermal stimulation was found to be also safe when using infrared irradiation with radiant exposure below 1 J/cm^2^^37^. In the brain, Chernov et al. showed for the first time that using less than 0.4 J/cm^2^ per pulse in 0.5 s-long 200 Hz trains can be safely used in rodents and squirrel monkeys^18^. In the human root nerve, thermal damage was first noted at a radiant exposure of 1.09 J/cm^2^^19^. In anesthetized mice, qualitative evidence on cell or tissue swelling, fragmented, lysed or ablated cells or displaced tissue was not found after using 250 μs pulses applied at 200 Hz during 500 ms long infrared (ƛ = 1,470 nm) radiation^38^. These reports underpin the importance of the targeted tissue, however, they all focus on testing the excitatory effect of pulsed-wave IR stimulation. Moreover, these studies did only apply superficial illumination of the tissue without giving information on deep-tissue stimulation. As Brown et al. points out, the in vivo safety assessment of infrared stimulation is important, as in vitro studies show higher damage thresholds^39^. Our in vivo study on deep tissue illumination reveals that suppression of cellular activity with continuous-wave infrared light coupled through a sharp-tip intracortical optrode is also an efficient and safe approach for neuromodulation in the central nervous system.

## Methods

### Optrode

#### Design and fabrication of sharp tip optrodes

The implantable optrode chip was designed to maximize the optical efficiency of the device while minimizing the tissue damage during insertion. Simulation results of our group have shown that for symmetric optrode tips the total internal light confinement has a considerable risk for α tip angles between 35.5–73.3°^24^. To reduce the reflection and maintain robustness of the chip, we defined tip angle as 2α = 30°. Since the shaft of the optrode acts as a mechanical carrier and a waveguide, the cross-section was not changed along the shaft to reduce propagation loss. A 12-channel linear array with 100 µm inter-site distance was aligned from 200 µm from the tip to measure neuronal activity modulated with intracortical IR illumination. The length, width and thickness of the shaft are 5 mm, 170 µm, and 200 µm, respectively.

All geometric parameters are scalable depending on the location and the density of the neuronal population to be stimulated and monitored. The optrode was made of p-type single-crystalline silicon by planar and bulk micromachining (MEMS technology) sufficiently detailed in^8,20^. The Pt wiring and contact material of the electrophysiological recording sites were realized using sputtered platinum patterned by standard photolithography and lift-off technology. Besides holding wire bonding pads to form electrical interconnection with an external electrical connector, a groove for optical fiber alignment was also fabricated applying deep reactive ion etching (DRIE). After DRIE the high surface roughness sidewalls of the silicon chip were polished with a mixture of HF:HNO_3_:H_3_PO_4_ in a ratio of 1:8:1 at 20 °C for 2.5 min to reduce the scalloping nature of the surface and to provide a smooth surface essential for a high efficiency of the waveguiding shaft^20,40^. The individual optrode chips then were bonded to a custom printed circuit board (PCB) designed for high quality acute implantation in rodent subjects. The PCB besides providing electrical connections helps the proper alignment and fixing of the optical fiber (core ø = 105 µm, 0.22 NA) which delivers IR light from an external source (LPSC-1550-FG105LCA-SMA pigtailed laser diode, Thorlabs Inc., USA). The rectangular (30 × 30 µm^2^) recording sites have additional porous Pt coverage (also called as platinum black or b-Pt) to reduce the impedance magnitude.

#### Characterization of optical heating

To characterize the operation of the optrode shaft as a waveguide, a custom setup for temperature measurement in water as a model liquid medium was established. The sharp tip optrode delivering the infrared light and a MEMS based calibrated platinum thermometer were immersed in a 2 ml polyethylene (PE) cylinder filled with water. More details on the thermometer and its calibration can be found in^20,28^. Microdrives holding each component of the setup provided an accuracy of 10 µm to align the thermometer with respect to the optrode tip. The perpendicular position of the axis of both tools were maintained throughout the experiments. IR irradiation absorbed in water and converted to local temperature rise of the medium was measured at different spatial coordinates along axis X and Y with 50 and 100 µm resolution, respectively. Direction parallel and perpendicular to the optrode axis were referred to as axes ‘x’ and ‘y’, respectively. IR light (λ = 1,550 nm) was delivered from a laser diode (LPSC-1550-FG105LCA-SMA, Thorlabs Inc., USA) controlled with Keithley 2611B SYSTEM SourceMeter (Keithley Instruments Inc, OH, USA). Temperature data were read out from the abovementioned thermometer by 4-wire resistance measurement of the Pt sensor realized by a Keithley 2100 6½-digit multimeter (Keithley Instruments Inc, OH, USA). A schematic drawing of this experimental setup can be seen on Fig. 2a in^8^.

First, this setup was used to determine the relationship between the supply current of the laser diode coupled to the optrode and the temperature elevation in the surrounding medium. The supply current of the light source was tuned between 60–440 mA by a Keithley 2611B SYSTEM SourceMeter (Keithley Instruments Inc, OH, USA). This calibration curve in Fig. 2E was recorded in a fixed position (at x = 200 µm and y = 0) and was used as a reference throughout the in vivo experiments. As written above in chapter ‘Suppression of neural activity', we probed the in vivo heating effect of IR irradiation with three different driving current levels of the laser: 200, 300 and 400 mA. According to our previous findings in^8^, based on the mentioned calibration curve in Fig. 2E, the estimation of the change in tissue temperature can be performed. The estimated temperature elevation achieved by the applied 200, 300 and 400 mA laser supply are + 2.04, + 2.98 and + 3.56 °C with the use of a sharp tip optrode, which was used during in vivo experiments.

The spatial distribution of the temperature around the sharp optrode tip was recorded between x = 0–1,600 µm at y = 0 position and y = –1,200– + 1,200 µm at x = 5|100|200 µm positions. Please note that some optical heating experiments with blunt tip optrodes applied a different IR light source. For more details see^8^. During each measurement, the laser was driven with a five-cycle-long square wave current signal with 50% duty cycle and 2 min period (1 min ON and 1 min OFF). The obtained temperature elevation induced by the absorbed IR light was calculated for each square wave cycle by averaging the last 254 data points (sampled at 25.45 Hz) of the plateau phase, then the average of these five values were considered as a single data point in both the calibration curve and in the spatial characteristics.

#### Characterization of electrophysiological recording sites

The recording sites of the sharp tip IR optrodes were investigated by electrochemical impedance spectroscopy (EIS). A three-electrode electrochemical cell was assembled in a Faraday cage and connected to a Gamry Reference 600 Potentiostat (Gamry Instruments, Warminster, PA, USA). The electrolyte was a 0.01 M room temperature solution of phosphate buffered saline (P4417, tablet diluted in 200 ml deionized water, Merck KGaA, Germany) in which a leakless miniature Ag/AgCl electrode (ET072-1, eDAQ Pty Ltd., Australia) and a Pt wire were immersed as the reference and counter electrodes, respectively. Platinum recording sites were used as working electrodes. The frequency range from 1 Hz to 10 kHz was swept. The amplitude of the excitatory voltage signal was 25 mV_RMS_.

The impedance magnitude of the recording sites was reduced by electroplating. A porous coverage of Pt was formed on top of the surface of the original sputter-deposited sites. The same Gamry instrument was used in galvanostatic mode. Another three-electrode electrochemical cell was also assembled in a Faraday cage. In this case, the electrolyte was a lead free 1 wt.% chloroplatinic acid solution (diluted from 8 wt.% chloroplatinic acid solution in H_2_O, Merck KGaA, Germany) with PVP (Polyvinylpyrrolidone, Merck KGaA, Germany) to improve the wettability of the sputtered Pt surfaces. The counter electrode was a Pt sheet which was positioned in parallel with the optrode to achieve similar even deposited layer thickness on each site. To the reference and working electrode connectors the same Ag/AgCl electrode and one of the recording sites were connected similarly to impedance spectroscopy. The porous layer was electroplated by maintaining current density of 10 mA/cm^2^ for 60 s. To control the potentiostat instrument—both in potentiostatic or galvanostatic mode—the Gamry Framework 7.8.4. software was used. Gamry Echem Analyst 7.8.4. and Microsoft Excel software were used to evaluate the results and visualize the impedance data in Bode plots.

### Surgery

Experiments were made in accordance with the Hungarian Act of Animal Care and Experimentation (1998, XXVIII) and with the directive 2010/63/EU of the European Parliament and of the Council of 22 September 2010 on the protection of animals used for scientific purposes. Experimental protocol was approved by the Medical Research Council (ETT) (license number PEI/001/2290-11/2015 for our in vivo experiments). Efforts were made to minimize the number of animals used. Rats were kept under a 12:12 h LD cycle (lights-on at 7:00 a.m.) in a temperature-controlled room at 22 ± 2 °C. Standard food-pellets and tap water were available ad libitum.

Our acute experiments were carried out on 5 male Wistar rats (Toxicoop, Budapest, Hungary) weighing between 300 and 440 g at the time of the surgery. The animals were anesthetized with urethane (1 g/kg; i.p.), then placed in a stereotaxic instrument (RWD Life Science; Shenzhen, China). Small craniotomies and durotomies were made over the left and right somatosensory cortices at AP -2 and 3 mm and L ± 3.0 mm, respectively. To record local field potential (LFP) activity, custom designed probes featuring twelve electrophysiological recording sites and a built-in light cable were inserted into the brain at a depth of 1,600 µm. After electrode insertion, we waited at least for 30 min before recording. An additional screw electrode implanted over the cerebellum served as a reference. Coordinates are based on the stereotaxic atlas of Paxinos and Watson^41^.

To analyze the effect of device geometry on neuronal survival with optical stimulation and without infrared irradiation (geometrical dummies), both blunt-tip and sharp-tip devices have been implanted on the contralateral side. Dummy devices were left in the tissue for the same time as fully operated devices before perfusion.

### Electrophysiology recordings and evaluation

Recordings were made with Intan RHD2132 16-channel amplifiers, connected to an RHD-2000 Evaluation Board. Heating was triggered by a square pulse generated by an NI-USB 6211 (National Instruments, TX, USA) data acquisition system. All signals were sampled at 20 kHz and synchronized through the analog input of the Intan system, which was connected to an x86 based PC.

Raw LFP channels were band pass filtered between 0.4–7 kHz, and multi-units were detected with an absolute threshold. The unit activity was combined from multiple neighboring channels, downsampled to 1 kHz and smoothed with a 10 ms moving average filter. This data was used for calculation of peri-stimulus time histogram (PSTH) of heating events.

### Optical stimulation protocol

To characterize the effect of IR stimulation performed by the sharp tip optrode device, three different stimulation protocols were tested. Each protocol was repeated 10–12 times. The laser diode with λ = 1,550 nm IR center wavelength (LPSC-1550-FG105LCA-SMA, Thorlabs Inc., USA) was driven by a Keithley 2611B SYSTEM SourceMeter (Keithley Instruments Inc, OH, USA). The applied heating protocols consisted of two sections: 2 min long stimulation onset (laser is switched on) was followed by 4 min long post-stimulus periods (laser is switched off). The aim of this longer pause in IR irradiation is to provide sufficient time for tissue temperature to return to its original value. Each onset of the laser diode was synchronized with trigger signals generated by an NI 6211 Data Acquisition Board (National Instruments, Austin, TX, USA). Estimation of the change in tissue temperature and thermal cross-section can be performed based on Fig. 2.

### Histology

Following the recordings, under deep anesthesia rats were transcardially perfused with 0.9% saline, followed by fixative containing 4% paraformaldehyde and 0.1 M phosphate buffer (PB). After perfusion brains were sliced to 50 µm thick coronal sections with a Leica VT1200S vibratome (Leica Biosystems, Wetzlar, Germany). Neuronal nuclei immunostaining (NeuN, Millipore, 1:3000) was used to visualize neuronal cell bodies. The immunostained sections were dehydrated using xylene and coverslipped with DePeX (Serva) for light microscopic analysis carried out by a Leica DM2500 microscope (Leica Microsystems, Wetzlar, Germany).

### ARRIVE statement

The authors declare that they did their best to follow the recommendations in the ARRIVE guidelines (see: https://arriveguidelines.org).

## Supplementary Information

Supplementary Information.

## Data Availability

The data sets generated during and/or analyzed during the current study are available from the corresponding author on reasonable request.
