# Supplementary material for: Histological and electrophysiological evidence on the safe operation of a sharp-tip multimodal optrode during infrared neuromodulation of the rat cortex
Source: Sci Rep. 2022 Jul 6;12:11434. doi: 10.1038/s41598-022-15367-4 (PMC9259743; doi:10.1038/s41598-022-15367-4)
Supplement: Supplementary file 1 — Supplementary Information. [file 41598_2022_15367_MOESM1_ESM.docx]

# Supplementary


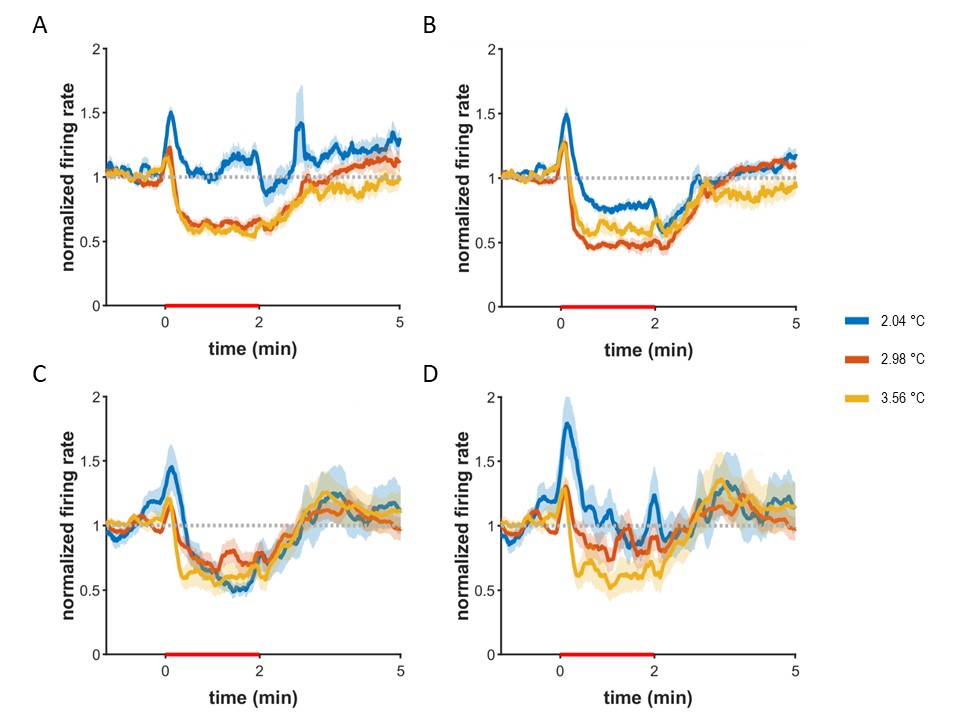


Supplementary Figure S1. Infrared light induced suppression of cortical neurons from the same cohort of rats in our experiments. Normalized mean firing rate traces based on multiunit activity. Panels A-D show the MUA response to the applied heating protocols in four individual rats (other than that presented in Figure 3). Red lines on horizontal (time) axes mark the presence of infrared illumination.
